# Taking a “Care Pathway/Whole Systems” Approach to Equality Diversity Inclusion (EDI) in Organ Donation and Transplantation in Relation to the Needs of “Ethnic/Racial/Migrant” Minority Communities: A Statement and a Call for Action

**DOI:** 10.3389/ti.2023.11310

**Published:** 2023-07-31

**Authors:** Alessandra Agnese Grossi, Gurch Randhawa, Nichon Esther Jansen, David Paredes-Zapata

**Affiliations:** ^1^ Center for Clinical Ethics, Department of Biotechnologies and Life Sciences, University of Insubria, Varese, Italy; ^2^ Department of Human Sciences, Innovation and Territory, University of Insubria, Como, Italy; ^3^ Institute for Health Research, University of Bedfordshire, Luton, United Kingdom; ^4^ Dutch Transplant Foundation, Leiden, Netherlands; ^5^ Donation and Transplant Coordination Section, Hospital Clínic, Barcelona, Spain; ^6^ Surgical Department, University of Barcelona, Barcelona, Spain; ^7^ Donation and Transplantation Institute Foundation, Barcelona, Spain

**Keywords:** organ donation, organ transplantation, migrants, ethnic minorities, inequities

## Abstract

International evidence shows variation in organ donation and transplantation (ODT) based upon a range of patient characteristics. What is less well understood is the impact of patient “ethnicity/race/immigration background,” as these terms are defined and intended differently across countries. We also know that these characteristics do not operate in isolation but intersect with a range of factors. In this paper, we propose a framework that seeks to clarify the definition of the key terms “ethnicity/race/migrant” and to review how these communities are operationalized across European studies about inequities in ODT. Further, patients and the public wish to see Equality Diversity Inclusion (EDI) approaches in their everyday lives, not just in relation to ODT. We propose a ‘care pathway/whole-systems’ approach to ODT encompassing culturally competent public health interventions for a) the prevention and management of chronic diseases, b) improvements in public engagement for the promotion of the culture of ODT and enhancements in end-of-life care, through to c) enhanced likelihood of successful transplant among migrant/ethnic minority communities. Our framework recognizes that if we truly wish to take an EDI approach to ODT, we need to adopt a more social, human and holistic approach to examining questions around patient ethnicity.

## Introduction

There is a plethora of evidence in most countries across the world showing variation in organ donation and transplantation (ODT) based upon a range of patient characteristics. These include—but are not limited to—age, gender, ethnicity, income, educational level, cultural beliefs, and religiosity [1–3]. As highlighted recently within important documents published by the European Kidney Health Alliance and the European Society for Organ Transplantation [4–6], the prevention and elimination of inequities related to these aspects is emergent in ODT in Europe. However, relative to other factors, what is less well understood is the impact of patient “ethnicity” and/or “race” and/or “immigration background,” as these terms are defined and intended differently across countries [7]. It is well known that these communities are highly heterogeneous as there can be great variations even within ethnic minority groups depending on the individual reasons for and circumstances of migration, the time elapsed since immigration, the number of generations they have spent in a given country (i.e., first- [foreign-born of foreign-born parents], second- [native-born of foreign-born parents], or even third-/fourth-generation migrants), immigration history of host countries, integration policies, availability of culturally competent healthcare services, and other such characteristics [8–11]. According to the European Commission, in 2022, 447.2 million inhabitants are living in the EU. Of these, 23.7 million are non-EU citizens (5.3% of the EU’s total population), and 37.5 million people were born outside the EU (8.4% of all EU inhabitants) [12].

Pilot studies which have sought to examine these aspects in relation to ODT have shown that “ethnicity” and/or “race” and/or “immigration background” do not operate in isolation but intersect with a range of other socioeconomic factors at the individual level, and with other factors at the interpersonal and societal level [13, 14]. Therefore, to improve data comparability in Europe and to enable the effective development and subsequent implementation of interventions against inequities, there is a need for consensus on how these communities are conceptualized and what data should be collected when research is performed on these populations in ODT [6, 8]. Besides, research suggests that patients and the public do not wish to see Equality Diversity Inclusion (EDI) approaches only in relation to ODT but in their everyday lives (i.e., by improved education for the prevention and early treatment of chronic conditions that have the potential to lead to organ disease or failure). Yet, only few if any public health interventions in ODT have been framed so [8, 15].

To fill these gaps, this study aims to clarify the definition of the key terms “ethnicity,” “race,” and “migrant” and to review how these communities are operationalized in studies addressing inequities in ODT in Europe. Second, it puts forward a proposal for a “care pathway/whole-systems” approach to ODT encompassing culturally competent public health interventions for a) the prevention and management of chronic diseases, b) improvements in public engagement for the promotion of the culture of ODT and enhancements in end-of-life care, through to c) enhanced likelihood of successful transplant among migrant/ethnic minority communities.

## “Ethnic/Racial/Migrant” Minority Communities: What Operationalization in Relation to Organ Donation and Transplantation Across Europe?

The terms “ethnicity,” “race” and “migrant” are often considered synonymous; however, they are not interchangeable. For instance, although they are interconnected, there are substantial specific features related to each that require consideration [7]. “Ethnicity” is defined as “the social group a person belongs to, and either identifies with or is identified with by others, as a result of a mix of cultural and other factors including language, diet, religion, ancestry, and physical features” [16] that are shared by individuals in the same group [7]. Similarly—although we acknowledge that “there is only one race—the human race,” to quote Rosa Parks (civil rights activist US [1913–2005])—but still, differently, “race” is “the group […] a person belongs to as a result of a mix of physical features such as skin color and hair texture, which reflect ancestry and geographical origins, as identified by others or, increasingly, as self-identified” [16]. In contrast, a “migrant” is “any person who is outside a State of which he or she is a citizen or national, or, in the case of a stateless person, his or her State of birth or habitual residence. The term includes migrants who intend to move permanently or temporarily, and those who move in a regular or documented manner as well as migrants in irregular situations” [17]. At the European level, the European Commission stresses that the immigrant category does not include persons who travel for tourism or business purposes and excludes intra-European Union mobility [18]. Research has noted that immigrant status cuts across the traditional social determinants of health and has the potential to amplify them at multiple levels which influence a person’s health. These include biological factors (i.e., age, sex, constitutional factors), individual lifestyle factors, social and community influences, living and working conditions, and the general socioeconomic, cultural, and environmental conditions [19]. Similarly, although it is well-established that ethnicity is a social construct that may vary over time within individuals and across generations, and according to political, cultural and societal features, an individual’s ethnicity can provide clinical clues that may be of value for medical purposes and for studying differences in populations that may be relevant to health. These include geographic origin and immigrant status, housing conditions and employment patterns, dietary habits, cultural and environmental factors, and genetic ancestry [20]. It follows that immigrant status diverges from ethnicity and race, but simultaneously includes elements of both. These features can be present at various intensities across the composition of different ethnic minority groups. However, while ethnicity in the United States (US) is mostly self-perceived, the operationalization of ethnicity in Europe is heterogeneous in that it is broadly defined by surrogate variables (i.e., country of birth, citizenship, former citizenship, etc.) [7]. Studies have stressed that lack of consensus surrounding these concepts extends to studies in ODT [6]. However, formal research regarding this aspect is lacking. To fill this gap, we reviewed studies assessing inequities among these communities at the different stages of the ODT process and confirmed that, in the European context, these populations are categorized in a heterogeneous fashion. Besides, we found that most, except few, studies are retrospective (i.e., not always adjusted for potential confounders and unable to determine the impact of the factors associated with ethnicity and immigrant status on inequities at the different phases of the ODT process), and focused on kidney transplantation (Supplementary Table S1). Studies performed in the UK categorize these individuals according to broad ethnicity categories such as Black, Asian/South Asian and other minority ethnic groups [8, 21–25]. The reason why the majority of studies does not provide any formal explanation of how ethnicity is determined is likely due to ethnicity data being available in either electronic patient records [25] or registry databases [24]. Yet, one study highlighted that, where ethnicity was not available, South Asian origin was derived by name screening [21], whereas another one explained that Black ethnicity was attributed to individuals that are genetically of Sub-Saharan African origin (mostly African Caribbean or West African) [23]. Studies from other European countries beyond the UK define these populations as first-generation migrants/ethnic minority populations based on country of birth alone [26–28], nationality and place of birth [29], citizenship and country of birth of patients and their families to allow the collection of more detailed data regarding migration history [30], and other unspecified factors [31]. Only one review article—which does define these populations as “migrants and ethnic minorities”—states that the data was missing by ethnic group [32], whereas another study classifies kidney transplant patients according to their racial background (i.e., Black) based on country of origin [33]. Most [34–37], but not all studies [38] of the pediatric patient population in the kidney transplant setting categorize these subjects as born of immigrant parents. The immigration background of parents is derived from at least one parent being a non-native speaker [36], one or both parents being born in a non-Western European country [37] or, more generically, being born of immigrant families [34, 35]. In contrast, one study classifies children based on their racial background according to the broader geographical area of origin [38].

Studies have noted that lack of consideration of within-group variations like cultures, language(s), religious affiliation, number of generations spent in the host country, time elapsed since immigration for first-generation migrants, socioeconomic status, and specific Human Leukocyte Antigen (HLA) types may lead to failure of considering the features with the potential to provide valuable insights into the heterogeneity of different ethnic minority groups and enable the development of more targeted interventions accordingly [8, 25, 39].

The European Public Health Association acknowledges the controversial definition and categorization of “ethnic minorities” in Europe. However, it stresses that, regardless of how these populations are defined and categorized, the features related to “ethnicity” are frequently rather independent of ‘immigrant status’ [40]. Besides, individuals who have migrated from other countries or who are from ethnic minorities in Europe are likely to experience similar health inequities [40, 41], biological features vary among ethnic groups [42], and individuals belonging to “visible” ethnic minorities may experience more significant inequities [43]. Therefore, we contend that both the features related to “ethnicity” (including country of origin or descent) and “immigrant status” (or immigration history) should be considered for the purposes of studies in relation to ODT. Additionally, the intersection with the multiple factors associated to each requires further research.

### Prevention and Treatment of Long-Term Conditions at Risk of Organ Disease or Failure

The World Health Organization suggests that 80% of non-communicable diseases including premature heart disease, stroke and diabetes may be prevented by intervening on risk factors (i.e., tobacco use, physical inactivity, unhealthy diet, and alcohol abuse) through behavior change interventions [44]. However, migrant and ethnic minority populations are disproportionately affected by chronic conditions like diabetes, hypertension, and obesity, and also communicable diseases such as hepatitis B and C [45, 46] which all have the potential to lead to end stage organ failure.

For example, in the United Kingdom (which has a longer-standing immigration history relative to other European countries), kidney dysfunction is a problem that disproportionately affects Black, Asian and Minority Ethnic individuals (BAME). Although BAME people represent only 11% of the UK population, they accounted for 24% of patients dependent on renal replacement therapy in 2017. BAME people are more likely to develop chronic kidney disease than white people, and those who have chronic kidney disease experience faster decline in kidney function than white people. There is some emerging evidence from the UK Biobank that HLA alleles may have an impact upon kidney function [47]. Understanding the genetic causes of kidney dysfunction in BAME people in the UK and elsewhere could play an important role in reducing these inequities [47]. Such as the case of genetic susceptibility to kidney disease, particularly for variants in the APOL1 gene that are associated with kidney disease. Research suggests that the presence of two of these APOL1 variants was significantly associated with increased progression of CKD candidates with sub-Saharan African ancestry, including African American and Caribbean populations [48].

Additionally, some important factors to consider is how mortality rates for patients undergoing dialysis differ by ethnicity. A study from the United States (US) exploring this aspect found substantial differences by ethnicity across the 50 states. After matched analyses for comparable age and risk factors, mortality risk no longer differed for Whites or Blacks but remained much greater for territory-dwelling Hispanics and Asians [49]. It should be emphasized that certain blood and HLA groups are common among certain ethnic minorities and rare among the proportionately more numerous Caucasian donors. In kidney transplantation, and particularly in the case of young recipients, poor HLA class II matching is more frequent for patients from certain ethnic minorities and unfortunately highly detrimental for graft outcome. Therefore, it is important to inform people of the interest of being all organ donors after their death, regardless of ethnicity and to better understand the implication of reduced HLA class II matching policies in kidney allocation and reconsideration of best practices to reduce inequalities while optimizing patient outcomes [50]. Since organ allocation rules are national and transparent, only the blood group and not the HLA group must be taken into account for liver, lung or heart transplants and access to transplants should be almost comparable for those patients actively registered on the waiting list (WL), regardless of their ethnic origin and whether or not they are migrants.

### Why the Shortage of Organ Donors

Shortage of *post mortem* organ donors in relation to the number of patients on the WL for transplantation is seen in many countries, although there are differences [51]. When taking a closer look at WLs, ethnic minorities appear to be disproportionately represented [8]. As illustrated previously, this is due to the high prevalence of chronic disease conditions among these populations [45]. For instance, data from Europe (including the United Kingdom) and North America show that many migrant and ethnic minority communities have a higher risk of developing end stage organ disease (especially kidney disease), are disproportionately represented in the patient population requiring renal replacement therapy and wait longer to receive a kidney transplant, compared with the “white” population [8, 52]. However, the number of post-mortem organ donors from migrant and ethnic minority communities is low for several reasons. First, there is a strong association between patient race/ethnicity and increased use of life-prolonging treatments, longer hospital stays, and intensive care units as a location of death. The Ethicus-2 study showed that end-of-life care practices vary among intensive care units worldwide [53]. On the other hand, the availability of tools to support end-of-life decision-making with patients and families from ethnocultural minority backgrounds is largely unknown [54]. Prolonged life treatments, religious beliefs and cultural perspectives, including language, can form barriers to discuss organ donation as part of end-of-life care.

Second, when looking at the organ donor register, for example, in the UK, more people identifying as white are present on the donor register than all other ethnic groups [55]. Reasons for the lack of sign up in these groups include lower donation knowledge, inferior likelihood to discuss donation and their wishes with family members, unacceptability due to religious beliefs and lack of trust in healthcare professionals.

Third, when donation is requested to a donor family the consent rate among ethnic minorities (blacks, Hispanics, and Asians) is lower than in whites [56]. As the authors’ stated, lower consent rates may be due in part to personal, cultural, or religious beliefs. However, portions of these differences are due to disproportionate miscommunication, misinformation, or lack of trust among migrant and ethnic minorities.

As a result, there is a need for end-of-life care which meets the needs of this group of patients. Providing end-of-life care to patients from different cultures is a challenge for services as there can be barriers to communication in the form of language, delegated decision-making within families, and reluctance to discuss about death [57–59]. It is important that solutions to EDI in end-of-life care are taken synchronously with developing culturally competent approaches to organ donation [57, 58].

### Likelihood of Transplant Accessibility and Successful Outcomes

There are substantial variations in kidney transplant incidence, prevalence, availability, accessibility, and quality worldwide, with the lowest rates evident in low- and lower-middle income countries. Understanding these inequities will inform efforts to increase awareness and the adoption of practices that will ensure that high-quality kidney transplant care is provided around the world [60].

Despite increasing interest in equitable healthcare, inequities in access to solid organ transplantation, especially among “ethnic/racial/migrant” minority patients are documented. In the UK, non-white ethnic minorities—mainly of Indian, Pakistani and Caribbean descent—comprise 11% of the population, 7% of organ donors, 35% of people awaiting a kidney transplant, and 21% of people who died on the WL. Norway has an increasingly diverse population. Many non-white migrant and ethnic minority groups, largely of Somali, Pakistani, Syrian, Iraqi and Eritrean descent, share many of the same risk factors for end stage kidney disease (ESKD), though currently protected by a younger age demographic. Little is known about ethnicity and organ donation in Norway because ethnicity data is not routinely collected, and where this is done only country of birth is recorded. Blood and tissue types differ between ethnic groups but are more often shared by close family members and people of the same ethnicity. Ideally, donors should be as diverse as the recipient population [32].

A review of inequities in access to heart, lung, liver, pancreas and kidney transplantation based on the social determinants of health (race, income, education, geography, insurance status, health literacy and engagement) in the US found that racial and ethnic minorities, women, and patients in lower socioeconomic status groups are less likely to be referred, evaluated, and registered on the transplant WL. Yet, the quality of the data describing these inequities was variable across the transplant literature and overwhelmingly focused on kidney transplantation [1], similar to studies in Europe (Supplementary Table S1)*.*

Beyond kidney transplantation, most studies on ethnicity-based outcomes in pancreas transplantation are from the United States, where healthcare delivery is predominantly insurance-based. There is no equivalent data from the United Kingdom, where the healthcare system is publicly funded. A recent article published in 2022 reported the first single center experience on ethnicity-based outcomes of pancreas transplantation from the United Kingdom. A retrospective analysis was performed of all patients who received pancreas transplantation (*n* = 171; Caucasians = 118/BAME = 53) from 2006 to 2020 (median follow-up = 80 months). Pancreas graft and patient survival were equivalent in both groups. BAME recipients had a higher prevalence of type-2 diabetes mellitus pretransplant (BAME = 30.19% vs. Caucasians = 0.85%, *p* < 0.0001), and waited for a similar time to transplantation once waitlisted, although pre-emptive simultaneous pancreas–kidney transplantation rate was higher for Caucasian recipients (Caucasians = 78.5% vs. BAME = 0.85%, *p* < 0.0001). Despite equivalent rejections and steroid usage, BAME recipients gained more weight (BAME = 7.7% vs. Caucasians = 1.8%, *p* = 0.001), but had similar HbA1c (functioning grafts) at 3-, 12-, 36-, and 60-months post-transplant [61].

There are several social determinants that need to be supported to both reduce the rates of organ failure, as well as increasing the rates of transplantation, particularly in at-risk populations. Some of these determinants overlap, including finances, transportation, psychosocial issues, and family support. However, given that uncontrolled high blood pressure and diabetes are the leading causes of chronic kidney disease, primary care infrastructure, access to culturally competent preventative health services, combined with health-disadvantaged lifestyles and environment lead to kidney failure often prevent people from remaining on the transplant WL [62] Intersecting patient-, provider- and healthcare system-related factors may have negative downstream effects on the subsequent phases of the transplant process, including access to the WL and, later in the timeline, transplant outcomes [52]. Late referral for transplantation is common among migrant and ethnic minority populations, especially among individuals who are younger, with diabetes, and with a higher degree of social deprivation [21]. Further, the health status during the waiting phase may be more impaired due to delayed access to appropriate care. Or, once transplanted, they cannot maintain the required lifestyle. It is likely that transplant outcomes could be improved in at-risk populations if the social determinants of health were addressed [63]. Besides, studies of patients who are in the transplant process suggest that some important steps are required to address the social determinants of health and race-related inequities in kidney transplantation. Education, community-based workshops, and awareness campaigns may increase the number of Black people who receive living and deceased donor organ transplantation. Similar to individuals who are already in the transplant process, it is likely that many patients on dialysis would be great transplant candidates if they had more education and support [64].

Collection of national surveillance data on early transplant steps, as well as routinely captured data on upstream social determinants of health—including the measurement of patients’ perceived discrimination [65] rather than race *per se*—is necessary to enhance understanding of the barriers to referral and evaluation. A multipronged approach (i.e., targeted and systemwide interventions, and policy change) implemented at multiple levels of the healthcare system will be necessary to reduce inequities in early transplant steps [66].

Further, although living donor kidney transplantation (LDKT) is the best renal replacement therapy for patients with ESKD providing survival advantages over dialysis and deceased donor kidney transplant, studies report diminished uptake of LDKT among migrant and ethnic minority populations [22, 24–26, 31]. This has the potential to lead to inferior outcomes among migrant and ethnic minority kidney transplant recipients [31]. A focus group study including 50 ESKD patients in the Netherlands explored modifiable hurdles to LDKT. They found that, although nearly all patients were in favor of LDKT (96%), multiple factors played a role in considering LDKT. These included inadequate patient education, impeding cognitions and emotions, restrictive social influences, and suboptimal communication. With regard to solutions to address the factors that influence equality in access to LDKT, they found that most patients (88%) were open to home-based group education about renal replacement therapy options. The study highlights the need for sensitivity and awareness of the influence of cultural factors on decision-making when discussing living donation with culturally diverse populations [67].

From 2013 to 2018, custom datasets from the United States Renal Data System and the United Network for Organ Sharing were merged to calculate the Kidney Transplant Equity Index (KTEI), defined as the number of minority patients transplanted at a center relative to the prevalence of minority patients with ESKD in each center’s health service area. Markers of socioeconomic status and recipient outcomes were compared between high and low KTEI centers. The KTEI is the first metric to quantify minority access to kidney transplant incorporating the prelisting ESKD prevalence individualized to transplant centers. KTEIs uncover significant national variation in transplant practices and identify highly equitable centers. This novel metric should be used to disseminate best practices for ESKD patients from ethnic minority groups and with inferior socioeconomic status [68].

## Taking a “Care Pathway/Whole Systems” Approach to Organ Donation and Transplantation

**FIGURE 1:**
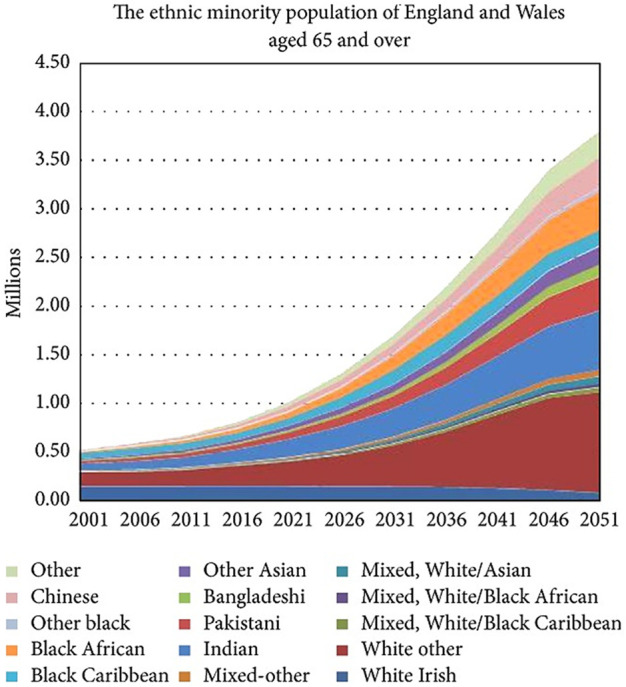
Ethnic minority population projections to 2051 in England and Wales. Source: Leveley 2010 [69].

**FIGURE 2:**
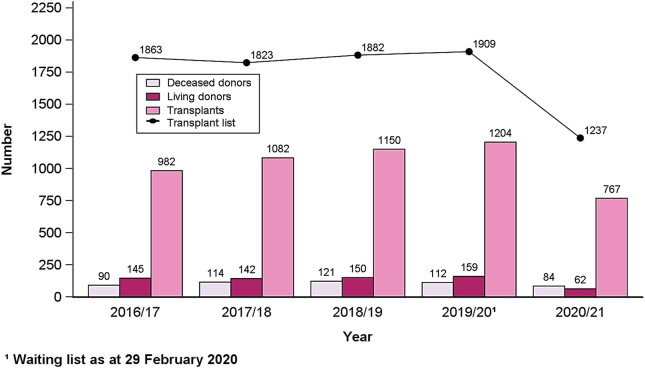
BAME deceased and living donors and BAME transplants in the United Kingdom, 1 April 2016 to 31 March 2021, and patients on the active transplant list at 31st March each year. Source: NHS Blood and Transplant [70].

Most countries are seeing life expectancy increase; however, this is not the case for all ethnic groups. As populations grow older, the demographics will change. For example, in the United Kingdom, most ethnic minorities comprise a younger demographic than the white British population. Yet, by 2051, this pattern will change with “Other White,” “Chinese,” “Other Asian,” “Indian,” “Other,” and “White Irish” alongside “White British” all being the ethnic groups with the highest representation aged 50 and over (Figure 1). By the mid-2050s, ethnic minorities will make up nearly half of the UK population. This illustrates the need for all countries with increasingly diverse ethnic populations to invest in the development of a whole-system approach to commissioning preventative and transplant services to satisfy the future requirements of an increasingly multi-ethnic older population [15]. For instance, a broad body of evidence shows that migrant and ethnic minority groups experience health inequities in many countries. This phenomenon can be observed in ODT services too, with significant variation in relation to demand for, access to, and waiting times for these services—especially among migrant and minority ethnic groups. Demand for transplantation can largely be reduced if there is a sustained commitment to public health interventions and culturally competent approaches implemented in the management of long-term conditions, recognizing the heterogeneity of diverse migrant and ethnic minority populations. Improved access to transplantation and reduced waiting times can be achieved if there are concerted and adequately resourced efforts to increase the number of organ donors from minority ethnic groups. This vision of equity and inclusion can only be realized by adopting a culturally competent approach to systems-wide working in organ donation in four core areas: public engagement; disease management; staff training; and transplant services [8]. The UK provides an interesting example where the recent positive trajectory in the numbers of organ donors and transplants from minority communities cannot be attributed to a single intervention but points to the emerging policy recognition, over the last 20 years, that ODT inequities exist in the UK, and that they need to be addressed by taking a systemic approach (Figure 2) [8].

Data show that transplant inequities persist for migrant and ethnic minority communities across the world, including the countries where these populations benefit from universal healthcare coverage in the European area (Supplementary Table S1). A system-wide approach—that is cognizant of the immediate urgency of increasing organ donors from all communities, whilst balancing the longitudinal approach to reduce the need for organ transplantation—is the only sustainable pathway to EDI. Many governments do not currently commission prevention and treatment services in this “system-wide” approach, leaving transplant services and prevention strategies on parallel paths, whereas points of convergence would be much more efficient and culturally competent in meeting population-health needs. There is scope to learn from initiatives such as Peer Educators, Living Transplant Initiative, and the Community Investment Scheme—whereby minority communities in the UK have been engaged in a discourse around both disease prevention, treatment options, and organ donation [8, 71]. The challenge is to evaluate such initiatives to understand the context in which they took place, the processes employed, as well as assessing the impact on organ donation rates. This will enable us to develop community-specific and context-specific approaches to EDI across the world.

## Limitations and Strengths

The main limitation of this work lies in the inconsistency of how migrant and ethnic minority populations are defined in European studies, which may result in a biased analysis. However, one of the goals of our study was precisely to examine how individuals from these communities are categorized in relation to ODT in Europe. Therefore, what seems to be a major limitation of the study is rather a strength in its ability to point out the need for clarification of terms and definitions to be addressed and harmonized across Europe, as noted by earlier European reviews on migrants’ health [72]. Further, the number of studies assessing inequities in ODT among migrant and ethnic minority populations residing in Europe is limited, and most have a retrospective design, challenging the opportunity to adjust outcomes for all the potentially confounding variables. Besides, although this is in line with prior reviews of inequities in organ transplantation [1], existing studies in Europe focus chiefly on ESKD and kidney transplant. Furthermore, we acknowledge that the macroeconomic context, different healthcare policies, social security systems and legislation varying among (and sometimes even within) countries may influence accessibility, quality and outcomes of care. The European Deprivation Index (i.e., a standardized measure of socioeconomic level across Europe for improved understanding and comparability of the mechanisms and causes of health inequalities) may be a valid measure for the development, implementation and assessment of new policies to address inequities across countries [73]. Future studies should include consideration of these factors. Besides, in contrast with previous studies from the United States, research examining deceased organ donation rates among migrant and ethnic minority populations are lacking.

This study has also several strengths, lying primarily in its ability to highlight the multiple gaps in research of inequities in ODT among migrant and ethnic minority populations in Europe. Further, it succeeds to stress on the need for engagement of a more coordinated European framework to enable harmonization of definitions across Europe. This may lead to improved consistency of data collection to allow better data reporting, interpretation and cross-country comparisons. Finally, while not denying cross-country variations, it points out the need for a shared, coordinated approach to these vulnerable communities.

## Conclusion

The prevention and elimination of inequities related to patient characteristics is currently emergent in ODT in Europe. What requires clarification is the impact of patient “ethnicity” and/or “race” and/or “immigration background” on inequities in ODT, as these terms are defined and intended differently across countries. We contend that both the features related to “ethnicity” and “immigrant status”—and the intersection with the multiple factors associated to each–should be considered to identify the modifiable factors for targeted interventions to improve equity in the ODT process for these populations. However, evidence shows that, in the European context, these populations are categorized in a heterogeneous fashion and that most, except few studies are retrospective (i.e., not always adjusted for potential confounders and unable to determine the impact of the factors associated with ethnicity and immigrant status on inequities at the different phases of the ODT process), and focused on kidney transplantation. Because inequities are documented among migrant and ethnic minority populations in the entire ODT process, a “care pathway/whole-systems” approach to ODT encompassing culturally competent public health interventions is needed for improved a) prevention and management of chronic diseases, b) public engagement for the promotion of the culture of ODT and enhanced end-of-life care, through to c) enhanced likelihood of successful transplant among migrant/ethnic minority communities. Our framework recognizes that if we truly wish to take an EDI approach to organ donation and transplantation—we need to adopt a more social, human and holistic approach to examining questions around patient ethnicity.

## Abbreviations

BAME, Black, Asian and Minority Ethnic; EDI, equality diversity inclusion; ESKD, end stage kidney disease; HLA, human leukocyte antigen; KTEI, kidney transplant equity index; LDKT, living donor kidney transplantation; ODT, organ donation and transplantation; UK, United Kingdom; US, United States; WL, waiting list.
