# Supplementary material for: Taking a “Care Pathway/Whole Systems” Approach to Equality Diversity Inclusion (EDI) in Organ Donation and Transplantation in Relation to the Needs of “Ethnic/Racial/Migrant” Minority Communities: A Statement and a Call for Action
Source: Transpl Int. 2023 Jul 31;36:11310. doi: 10.3389/ti.2023.11310 (PMC10437067; doi:10.3389/ti.2023.11310)
Supplement: Supplementary file 1 [file Table1.pdf]

Supplementary Table S1. Operationalization of ethnicity among studies of inequities in the process of organ donation and transplantation in Europe

	Study ID	*Study type*	*Country*	*Age-group*	*N*	*ODT setting*	*Definition of target group*	*Operationalization of ethnicity*	*Main findings*
Organ donation authorization	Randhawa et al. 2022 (8)	Review	UK	NS	NS	NS	BAME (Black, Asian, Other minority ethnic groups)	Broad ethnicity categories	People from minority ethnic communities underrepresented as eligible deceased donors, actual deceased donors, and opt-in on the NHS Organ Donor Register compared to the UK population
	Bhopal et al. 2019 (31)	Review	Norway	NS	NS	NS	Migrants and ethnic minorities	NS (authorization data by ethnic group was missing)	1/3 of refusal to deceased organ donation (but no data is available of ethnicity). Live organ donation is rare among migrant and ethnic minority groups
Waiting-list accessibility	Udayaraj et al. 2010 (20)	Incident cohort study (1997-2004)	UK	Adult	11,299 (9,602 White, 1,178 South Asian, 519 Black)	Kidney Tx	Ethnicity (White, South Asian, Black)	Broad ethnicity categories (for Pts with missing ethnicity data, South Asian origin was derived by name screening)	No major differences in waitlisting were detected by ethnicity
	Cantrelle et al. 2006 (28)	Retrospective cohort survey (1996-2003)	France	Pediatric and adult	18,595 (16,203 French Mainland, 704 FOT, 123 Greek, 261 Italian, 274 Other European, 693 North African, 337 SSA)	Kidney Tx	Non-French (Mainland French, FOT, Greek, Italian, Other European, North African, SSA)	Derived from nationality and place of birth	French residents of FOT and Pts from SSA waited significantly longer than other Pts
Transplant accessibility/likelihood	Grossi et al. 2022 (25)	Retrospective cohort study (2010-2020)	Italy	Adult	24,174 (21,624 EU-born, 606 Eastern European-born, 1944 non-European-born)	Kidney Tx	First-generation migrants (non-EU-born)/ethnic minorities	Derived from country of birth	Non-European-born Pts were less likely to receive LDKT but equally likely to receive DDKT and to be withdrawn from WL relative to non-EU-born and EU-born and

									Eastern-European-born Pts
	Khalil et al. 2022 (24)	Retrospective cohort study (single center) (2007-2020)	UK	Adult	1,940 (1,255 White, 421 South Asian, 135 Black, 129 Other)	Kidney Tx	Ethnicity (White, Black, South Asian [also referred to as Indo-Asian] and "Other")	Pre-determined ethnicity classifications, as obtained from electronic patient records	Individuals who are non-White or live in socioeconomically deprived areas have a reduced likelihood of receiving LDKT
	Oztek-Celebi et al. 2019 (35)	Cohort study (single-center) (2008-2013) and survey	Austria	Pediatric	77 (45 children of non-immigrant and 32 from immigrant families [10 former Yugoslavia, 10 Turkey, 10 from other countries])	Kidney Tx	Immigrant children	Family's descent classified as immigrant if the parents of the Pt were first-generation migrants (based on non-German mother tongue of at least one parent)	Migration-related inequities (information delay, limited communication, low knowledge levels, and self-reported conflicting beliefs) did not translate into inferior LD rates
	Wu et al. 2017 (21)	Prospective study (2011-2013)	UK	Adult	2,055 (1,692 White, 199 Asian, 129 Black, 31 Other)	Kidney Tx	Ethnicity (White, Asian, Black, Other [Chinese and mixed origin])	Broad ethnicity categories	Significant reduction in the likelihood of LDKT for Pts of Asian and Black ethnicity
	Tjaden et al. 2016 (37)	Retrospective cohort study (2006-2012)	Belgium, Czech Republic, the Netherlands, Portugal, Slovakia, Switzerland, UK	Pediatric	1,134 (868 White, 59 Black, 116 Asian, 91 other racial groups)	Kidney Tx	Racial background (White, Black, Asian [South-East and North-East Asian], "other" [Arabic-Middle Eastern, North African, and mixed])	Broad geographical area of origin	Black and Asian Pts were less likely than white Pts to receive first kidney Txt in the first 3 years after initiating RRT
	Tromp et al. 2012 (36)	Prospective study (multi-center) (2007-2011)	Netherlands and Belgium	Pediatric	119 (78 native-born and 41 immigrant children)	Kidney Tx	Immigrant children	Children with one or both parents who were born in non-Western European countries	Immigrant children had lower rates of preemptive Tx, spent more time on dialysis before Tx, received grafts from younger donors and median cold ischemia time was longer

	Udayaraj et al. 2012 (23)	Retrospective cohort study (1997-2004)	UK	Adult	12,282 (10,487 White, 1,243 South Asian, 552 Black)	Kidney Tx	Ethnic minority patients (White, South Asian, Black)	Pre-determined ethnicity classifications, as obtained from electronic registry data	Socially deprived and younger ethnic minority patients have lower probability of LDKT
Transplant outcome	Grossi et al. 2020 (26)	Retrospective cohort study (2010-2015)	Italy	Adult	6,997 (6,346 EU-born, 161 Eastern European-born, 490 non-European-born)	Kidney Tx	First-generation migrants (non-EU-born)/ethnic minorities	Derived from country of birth	Non-EU-born KTRs from non-European countries had worse eGFR decline after the first post-Tx year
	Bucher et al. 2019 (29)	Retrospective study (single center) (2007-2015)	Germany	Adult	358 (291 with no migration history and 67 with migration history)	Liver Tx	Migration status	Derived from citizenship and country of birth of Pts and their families	There were differences concerning age at Liver Tx and prevalence of underlying diseases. No statistical differences in re-Tx rates, overall survival, or other negative outcomes
	Tjaden et al. 2016 (37)	Retrospective cohort study (2006-2012)	Belgium, Czech Republic, the Netherlands, Portugal, Slovakia, Switzerland, UK	Pediatric	1,134 (868 White, 59 Black, 116 Asian, 91 other racial groups)	Kidney Tx	Racial background (White, Black, Asian [South-East and North-East Asian], “other” [Arabic-Middle Eastern, North African, and mixed])	Broad geographical area of origin	Tx survival rates were similar among racial groups
	Laging et al. 2014 (30)	Retrospective cohort study (single center) (2000-2010)	Netherlands	Adult	1,338 (977 European and 361 non-European)	Kidney Tx	Ethnic minorities (i.e. non-European) (African, Arabian, Asian, European, Turkish)	NS	SES and ethnicity did not have a significant influence on graft and Pt survival but inferior uptake of LDKT may affect outcomes
	Oztek-Celebi et al. 2011 (33)	Retrospective study (single center) (1978-2007)	Austria	Pediatric	196 (148 native and 48 immigrant)	Kidney Tx	Immigrant children	Children from immigrant families	Pt and graft survival, rejection-free survival, and frequency of acute rejection episodes

									were not influenced by migration status
	Ng et al. 2010 (22)	Retrospective study (single center) (1995-2006)	UK	Adult	555 (50 Black, 505 non-Black)	Kidney Tx	Blacks	Broad ethnicity categories (individuals genetically of SSA origin [mostly African Caribbean or West African])	Independent of CYP3A5 expresser status, Black KTRs had poorer long-term outcomes relative to those from other ethnic groups
	Mérida et al. 2009 (27)	Retrospective study (single center) (1996-2006)	Spain	Adult	76 (27 migrants and 49 Caucasian Pts from Spain)	Kidney Tx	Emigrants from Africa (Morocco, Guinea, Nigeria)	Derived from country of birth	Identical Pt and graft survival. African Pts needed higher doses of Tacrolimus and experienced more rare opportunistic infections
	Oztek-Celebi et al. 2009 (34)	Retrospective study (single center) (1997-2005)	Austria	Pediatric	59 (42 native and 17 immigrant)	Kidney Tx	Immigrant children	Children from immigrant families	No effect of migration status on the outcome of Kidney Tx
	Pallet et al. 2005 (32)		France	Adult	1,092 (952 Caucasian and 140 African European [39 SSA and 101 Caribbean]	Kidney Tx	Racial background (African Europeans [Pts from Africa and Caribbean])	Derived from country of birth	No statistically significant difference in allograft and Pt survival rates

DDKT, Deceased Donor Kidney Transplant; FOT, French Overseas Territories; KTR, Kidney Transplant Recipient; LD, Living Donation; LDKT, Living Donor Kidney Transplant; NS, Not Specified; Pt, Patient; RRT, Renal Replacement Treatment; SSA, Sub-Saharan Africa; Tx, Transplant; UK, United Kingdom; WL, Waiting List
